# Efficient and Rapid *C. elegans* Transgenesis by Bombardment and Hygromycin B Selection

**DOI:** 10.1371/journal.pone.0076019

**Published:** 2013-10-09

**Authors:** Inja Radman, Sebastian Greiss, Jason W. Chin

**Affiliations:** Medical Research Council Laboratory of Molecular Biology, Cambridge, United Kingdom; University of North Carolina at Chapel Hill, United States of America

## Abstract

We report a simple, cost-effective, scalable and efficient method for creating transgenic *Caenorhabditis elegans* that requires minimal hands-on time. The method combines biolistic bombardment with selection for transgenics that bear a hygromycin B resistance gene on agar plates supplemented with hygromycin B, taking advantage of our observation that hygromycin B is sufficient to kill wild-type *C. elegans* at very low concentrations. Crucially, the method provides substantial improvements in the success of bombardments for isolating transmitting strains, the isolation of multiple independent strains, and the isolation of integrated strains: 100% of bombardments in a large data set yielded transgenics; 10 or more independent strains were isolated from 84% of bombardments, and up to 28 independent strains were isolated from a single bombardment; 82% of bombardments yielded stably transmitting integrated lines with most yielding multiple integrated lines. We anticipate that the selection will be widely adopted for *C. elegans* transgenesis via bombardment, and that hygromycin B resistance will be adopted as a marker in other approaches for manipulating, introducing or deleting DNA in *C. elegans*.

## Introduction

To create transgenic *C. elegans* DNA may be introduced by microinjection [1], [2] or microparticle bombardment. While simple microinjection leads to extrachromosomal arrays that are lost over time, making it impossible to grow large populations, microparticle bombardment can lead to the creation of stable integrated strains [3], [4].

Although the microparticle bombardment method is widely used to generate transgenic *C. elegans*, it has several limitations including: i) the need to use specialized strains with a mutant background - to allow phenotypic isolation of transformants, ii) slow and labor intensive procedures for mutant isolation, iii) the requirement to handle large numbers of worms, and iv) loss of the transgenic array for non-integrated lines, v) the isolation of few, if any, transgenic lines from a given bombardment, and vi) the isolation of few, if any, integrated transgenics from a single bombardment. The use of antibiotic resistance genes and their corresponding antibiotics for selection of transgenic *C. elegans* in biolistic bombardments might in principle address many of these challenges.

Recently, the selection of transgenic worms created by microinjection was reported using antibiotic resistance markers and selection for transformation by growth on antibiotics. Two antibiotics and their corresponding resistance genes were tested: i) G418 was used in combination with a neomycin resistance gene, and ii) puromycin, which required the use of a detergent (Triton-X 100) to permeabilize the worms for the antibiotic to be effective, was used in combination with a puromycin resistance gene [5], [6]. The use of these antibiotic selection approaches for isolating transgenics from biolistic bombardments was not reported; furthermore, G418 selection was reported to be unsuccessful for selecting transformants via bombardment [6].

We reported the successful selection of transgenics following biolistic bombardment using a different antibiotic, hygromycin B, and the hygromycin B phosphotranspherase gene – encoding a kinase that inactivates hygromycin by phosphorylation – to select transgenic *C. elegans*, as part of our ongoing efforts to develop and apply methods to expand the genetic code of cells and animals[7]–[9]. Subsequently, the extensive characterization of a method for biolistic bombardment and enrichment of transgenics using a two-antibiotics plus detergent (puromycin, G418 and Triton-X 100) and a fluorescent protein gene, was reported [10]. Following bombardment, the dual antibiotic plus detergent method requires the removal of adults- which are not sufficiently sensitive to the antibiotic- by repeated gravity sedimentation, resuspension of the L1s and growth for 4 days in liquid media containing 0.5 mg ml^−1^ puromycin and G418 and 0.1% Triton-X 100 to enrich for transgenics. The antibiotic and detergent treatment is not sufficient to directly select transgenics and a further step, in which fluorescent transformants are manually isolated from a background of wild type animals, is required [10].

Antibiotic based selection has the potential to improve traditional bombardment approaches. However in the published dual antibiotic plus fluorescence approach 25% of all the bombardments in *C. elegans* reported fail to yield *any* transmitting transgenics. Moreover, in approximately half of the cases examined no integrated strains were identified using this approach. In most cases one independent line is isolated from a bombardment, and the maximum number of independent lines isolated by this approach is unknown. Here we develop and characterize a simple, rapid and cost-effective transformation and selection protocol to generate transgenic *C. elegans* using biolistic bombardment and hygromycin B selection and demonstrate the advantages of this approach.

## Materials and Methods

### Worm Strains and Maintenance

For bombardment either wild type *C. elegans* (N2 Bristol strain) or strains carrying the *smg-2*(e2008) allele were used. Worms were grown under standard conditions on NGM agar plates (1×NGM) [11] seeded with *E.coli* OP50. Post-bombardment selection was performed on 3×NGM agar plates (based on the recipe for 1×NGM, but with 7.5 g/l peptone, 0.1 mM CaCl_2_, 0.5 mM MgSO_4_) seeded with *E. coli* HB101, which give thicker bacterial lawns. Synchronized cultures for bombardments were grown in liquid medium containing: S-Basal (50 mM KPO_4_, pH 6.0, 100 mM NaCl, 5 mg/L cholesterol), 10 mM potassium citrate (pH 6.0), trace metals solution (50 µM disodium EDTA, 25 µM FeSO_4_×7H_2_O, 10 µM MnCl_2_×4H_2_O, 10 µM ZnSO_4_×7H_2_O, 1 µM CuSO_4_×5H_2_O), 3 mM CaCl_2_, 3 mM MgSO_4_, supplemented with Antibiotic-Antimycotic 100× (GIBCO Life Technologies). Worms in liquid culture were fed with concentrated *E. coli* HB101 pellets.

### Microscopy

Worm populations on NGM plates were imaged using a Rolera Bolt camera (QImaging) mounted on a Leica M165FC fluorescent stereo microscope with 16.5∶1 zoom optics. When acquiring fluorescence images, plates were cooled to 4°C before imaging to reduce the movement of the animals and allow for longer exposure times.

Confocal images were acquired using a Zeiss 780 UV confocal inverted microscope equipped with a 488 nm laser to image GFP and a 561 nm laser to image mCherry. Animals were washed off plates, anaesthesized using 0.2 mM levamisole and mounted on 3% agar pads.

### Survival Assays

Survival of N2 animals in the presence of various concentrations of hygromycin B was tested on NGM plates seeded with *E.coli* OP50. Worms were synchronized by bleaching and 300 (low density) or 3000 (high density) L1 larvae were added to seeded 6 cm plates and immediately treated with the indicated amounts of hygromycin B (diluted to 200 µl in M9 buffer). Plates were then incubated at 20°C and scored for L4 larvae after 40 hours and for adult worms after 60 and 90 hours. The experiment was performed in triplicate.

Survival of transgenic animals and selection efficiency was assayed by mixing 10 transgenic L1 larvae expressing both the hygromycin B resistance gene and GFP with 1000 or 10000 non-transgenic wild type L1s on a 6 cm seeded NGM plate. After transfer to the plate, hygromycin B was immediately added to a final concentration of 0.3 mg ml^−1^. The number of surviving adults was scored after 90 hours of growth at 20°C.

### Bombardment Protocol

Biolistic bombardment was performed using a He-1000 apparatus (Bio-Rad). The protocol was based on previously published protocols using rescue of the *unc-119* mutant phenotype as selection marker [3], [4].

Bombardments were performed with synchronized populations of worms at the late L4/young adult stage using gold beads and rupture discs as indicated. The best efficiencies were achieved with 0.3–3 µm gold beads (microcarriers) (ChemPur) and 1100 psi rupture discs (Bio-Rad).

To synchronize worms, the animals were grown until the culture contained a large number of adults, at which point feeding was stopped to induce growth arrest of any larvae hatched subsequently. After 1–2 days L1 larvae were harvested through removal of older animals by sedimentation and subsequent centrifugation of the supernatant for 4 min at 800 g and 4°C. The L1 larvae were used to start a fresh synchronized culture which was used for bombardment when the animals had reached the late L4/early adult stage.

For each bombardment 6 mg of gold beads were coated with 10 µg of DNA essentially as previously described [4]. Linearized DNA was not purified prior to coating, except when otherwise indicated. For co-bombardment of more than one vector, a minimum of 1 µg of resistance plasmid was used with 9 µg of co-bombarded plasmid(s).

60 mg of mixed beads (0.3–3 µm, ChemPur) were prepared for 10 bombardments (6 mg of beads/bombardment). The beads were first washed by vortexing for 30 min in 70% HPLC-grade ethanol (Sigma-Aldrich)**,** pelleted briefly and then washed 3×with sterile H_2_0 (for every wash, beads were vortexed for 1 min, soaked for 1 min, and spun down briefly). The beads were finally resuspended in 1 ml of 50% glycerol, and stored for a maximum of two weeks at 4°C if not immediately used.

For coating, 100 µl of beads in 50% glycerol (6 mg of beads) were transferred to DNA LoBind tubes (Eppendorf), centrifuged briefly, the supernatant removed and the beads resuspended in the DNA mixture, followed by the immediate addition of 20 µl fresh spermidine solution (100 mM in H_2_0) and 50 µl CaCl_2_ (2.5 M in H_2_0), with brief vortexing after each addition. The samples were then either incubated on ice for about 30 minutes with occasional resuspension or vortexed gently at 4°C. The beads were then washed once with 300 µl 70% HPLC-grade ethanol, once with 1000 µl 100% HPLC-grade ethanol and then resuspended in 140 µl 100% HPLC-grade ethanol and kept on ice until bombardment. We found that it is possible to store the DNA coated beads in 100% ethanol at −20°C at least over night without reducing transformation efficiency. Biolistic macrocarriers (Bio-Rad) and rupture discs (Bio-Rad) were washed in isopropanol (Sigma-Aldrich) and left to dry. The macrocarriers were then placed into a hepta-adaptor and 20 µl of DNA coated beads in ethanol were spread onto each macrocarrier. The hepta-adaptor with macrocarriers was then placed into the vacuum chamber of the He-1000 biolistic bombardment apparatus under vacuum until all ethanol had evaporated.

A Biolistic Hepta Stop Screen (Bio-Rad) and rupture disc were added and the adapter was mounted into the instrument. The plate with worms was placed at a distance of 6 cm from the stopping disk and the worms bombarded at 27.5 inches Hg vacuum.

### Hygromycin B Selection

After bombardment, the animals were left to recover at either room temperature or 20°C for 30 min to 1 h. The worms were then either washed off the bombarded plate and transferred to 10–20 selection plates (3×NGM, seeded with *E. coli* HB101) or alternatively cut into chunks and the chunks then placed onto the surface of the selection plates. The animals were left to lay eggs for 2–3 days, at which point the plates were generally full of very young F1 larvae. Hygromycin B was then added onto the surface of the plates.

Selections were performed by adding hygromycin B (>85% pure, Invivogen) onto the surface of the plates to a final concentration of 0.3 mg ml^−1^ hygromycin B, without the addition of any permeabilizing agents. The final hygromycin B concentration was calculated taking into account the total volume of the agar in the plate. To help spread the hygromycin solution evenly and to reduce post-bombardment contamination, we diluted the hygromycin B with several other antibacterial and antifungal solutions. The standard ‘selection mixture’ added to a single 9 cm plate was: 100 µl hygromycin B (>85% pure hygromycin B, 100 mg/ml, InvivoGen), 600 µl Antibiotic-Antimycotic 100× (GIBCO Life Technologies), 80 µl Amphotericin B 250 µg/ml (GIBCO Life Techologies), 20 µl 1000×Kanamycin solution (final concentration 50 µg/ml, Kanamycindisulfate, Merck). Selection mixture was added also to a control plate of non-transformed worms. In cases where the food on the selection plates was depleted at the time of hygromycin B addition, we added 100–200 µl of the concentrated bacteria used for growing liquid cultures 1–2 days after adding hygromycin B. Plates were then screened for transformants after a minimum of 4–7 days, at which time lines with high transmission rates were beginning to form populations. Plates that appeared to contain no transgenic worms were rescreened after a further 1–2 weeks.

We note that dauer larvae generally appeared to be resistant to hygromycin B. They were, however, unable to exit the dauer stage in the presence of antibiotic even when the population density was low and food was abundant. The presence of dauer larvae did therefore not negatively influence selection efficiency.

### Plasmid Construction

We created a 3-way Gateway pEXPR vector (SG120) carrying the hygromycin B resistance gene hygromycin B phosphotranspherase (*HygR*), which inactivates hygromycin B by phosphorylation, to be used for co-bombardment with any other vector of interest when creating transgenic strains [12]. For ubiquitous and strong expression of the gene, a 2.8 kb fragment upstream of *rps-0* was used [13].

### New Improved *HygR* Plasmids

In order to reduce the number of co-bombarded vectors, we created a pDEST Gateway vector where the Gateway cloning cassette is free for cloning purposes and the *HygR* resistance cassette was introduced into the pDEST vector backbone. Also, two multiple cloning sites, MCS1 (*KasI*/*NarI*/*SfoI*_*SpeI*) and MCS2 (*KpnI*_*NheI*_*AvrII*_*AscI*), were introduced upstream and downstream of the *HygR* cassette, to facilitate vector linearization (Figure S2 in File S1, Table S3 in File S1). The resistance cassette (P*rps-0::HygR*::*unc54*) was assembled by overlap extension (OE) PCR using primers WP21, WP64, WP65, WP68, WP23 and WP39. All PCR reactions were carried out using Phusion Polymerase (Finnzymes). The PCR product was then recombined into the pDONR P4-P1R Gateway vector (IR84). The resistance cassette was amplified from IR84 using primers WP191 and WP195, and the pDEST R4-R1 plasmid backbone was amplified using primers WP189 and WP190. Both PCR products were digested with *SpeI* and *AscI* and ligated to create the new pDEST variant carrying the resistance cassette in the backbone (IR95).

To reduce the size of our constructs, we tested a much shorter version of P*rps-0* than the one previously reported [7], [13] (0.8 kb instead of 2.8 kb) and confirmed that it is fully functional *in vivo*. To increase expression levels of the resistance gene, we codon optimized the gene and introduced artificial introns [14] (resistance cassette assembled by PCR using primers WP62, WP64, WP66, WP69, WP23 and WP48, recombined into pDONR 221 to give IR87; resistance cassette amplified from IR87 using WP193 and WP195 to create a pDEST IR98). We furthermore created versions of the resistance vector co-expressing a fluorescent marker, GFP or mCherry, from the same promoter as the resistance gene. The resistance –fluorescence operon was assembled by overlap extension (OE) PCR using primers WP43 (WP62), WP64, WP65 (WP66), WP127 (WP76), WP77, WP80 (WP82), WP44 (WP78), WP84 (WP85), WP23 and WP48. The PCR product was then recombined into the pDONR 221 Gateway vector (IR83, IR88, IR89, IR90). The operon cassette was amplified from these vectors using primers WP191 (WP193) and WP195, digested with *SpeI* and *AscI* and ligated with the pDEST digested backbone (described above) to create the new pDEST resistance vectors carrying a resistance – fluorescence operon (IR99– IR105) (Figure S2 in File S1, Table S3 in File S1).

## Results

**Figure 1:**
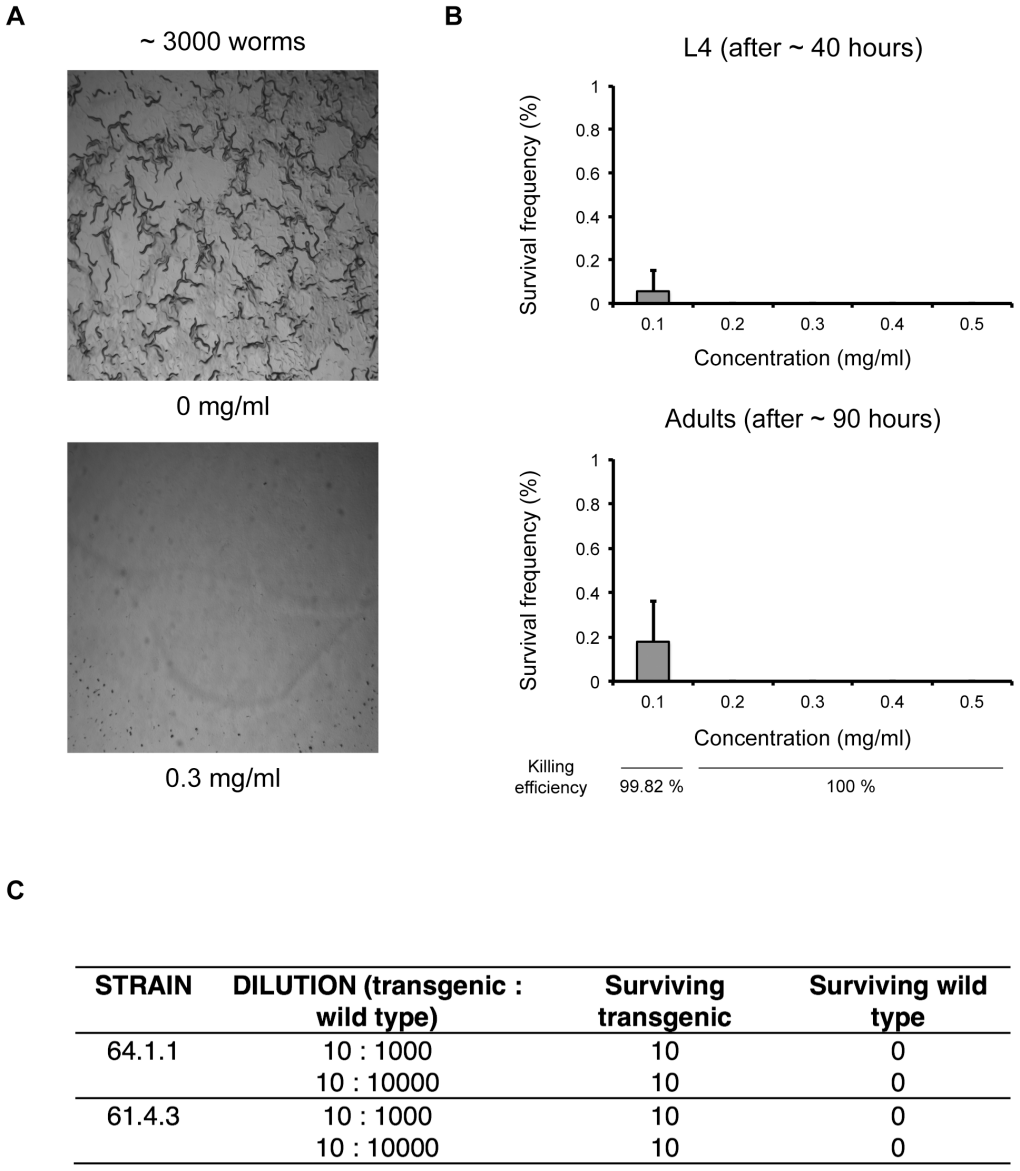
Survival of *C. elegans* in the presence of hygromycin B. a) 3000 synchronized L1 larvae were transferred to seeded 6 cm NGM plates and immediately treated with hygromycin B. Images of control and treated plates were acquired 40 hours later. b) Survival assay on hygromycin B dilution. 3000 synchronised L1 larvae were plated on seeded 6 cm plates and immediately treated with hygromycin B at the specified final concentrations. The plates were scored for L4 larvae after 40 hours and for adults after 90 hours. The experiment was performed in triplicate, error bars represent standard deviation. c) Selection of transgenic animals on hygromycin B. 10 synchronized L1 larvae carrying a hygromycin B resistance gene and expressing GFP/mCherry were mixed with the indicated number of wild type L1 larvae on a seeded 6 cm plate and immediately treated with hygromycin B (0.3 mg ml^−1^). The number of adult transgenic (fluorescence-positive) and non-transgenic (fluorescence-negative) animals was scored after 90 hours. The experiment was performed with two independent hygromycin B resistant transgenic strains.

We first investigated the efficiency of hygromycin B for killing wild-type *C. elegans*. We demonstrated that addition of low concentrations of hygromycin B to worms on the agar is sufficient to kill all L1 wild-type worms. For high worm densities (3000 worms per 6 cm plate) 0.2 mg ml^−1^ hygromycin B is sufficient to kill all non-transgenic worms (Figure 1, Figure S1 in File S1, Table S1 in File S1), at lower densities (300 worms per 6 cm plate) 0.1 mg ml^−1^ hygromycin B is sufficient to kill all non-transgenic worms (Figure S1 in File S1, Table S1 in File S1, Supplementary Note in File S1).

To demonstrate that the hygromycin B phosphotranspherase gene can rescue survival on hygromycin B we created several constructs (Figure S2 in File S1, Table S2 in File S1, Table S3 in File S1) for strong and ubiquitous expression of hygromycin B phosphotransferase from the P*rps-0* promoter in *C. elegans*. Final constructs used a *C. elegans* optimized *HygR* gene and a minimal P*rps-0* promoter to minimize vector size. We have created several Gateway compatible vectors carrying the *HygR* in the backbone, both with and without co-expressed fluorescent markers to quickly and easily generate constructs bearing genes of interest for bombardment. The vectors contain additional unique restriction sites, which should ensure that at least one site will be compatible with any given insert and thus facilitate linearization (Figure S2 in File S1, Table S2 in File S1, Table S3 in File S1). We demonstrated that transgenic worms bearing *HygR* are resistant to hygromycin B up to at least 1 mg ml^−1^ (data not shown) [7]. Moreover, we demonstrated in a model selection that when a small number of transgenic worms were placed in a 10^3^ fold excess of wild type worms and exposed to hygromycin B the wild type worms were all killed off while the transgenic worms all survived and developed (Figure 1c).

**Figure 2:**
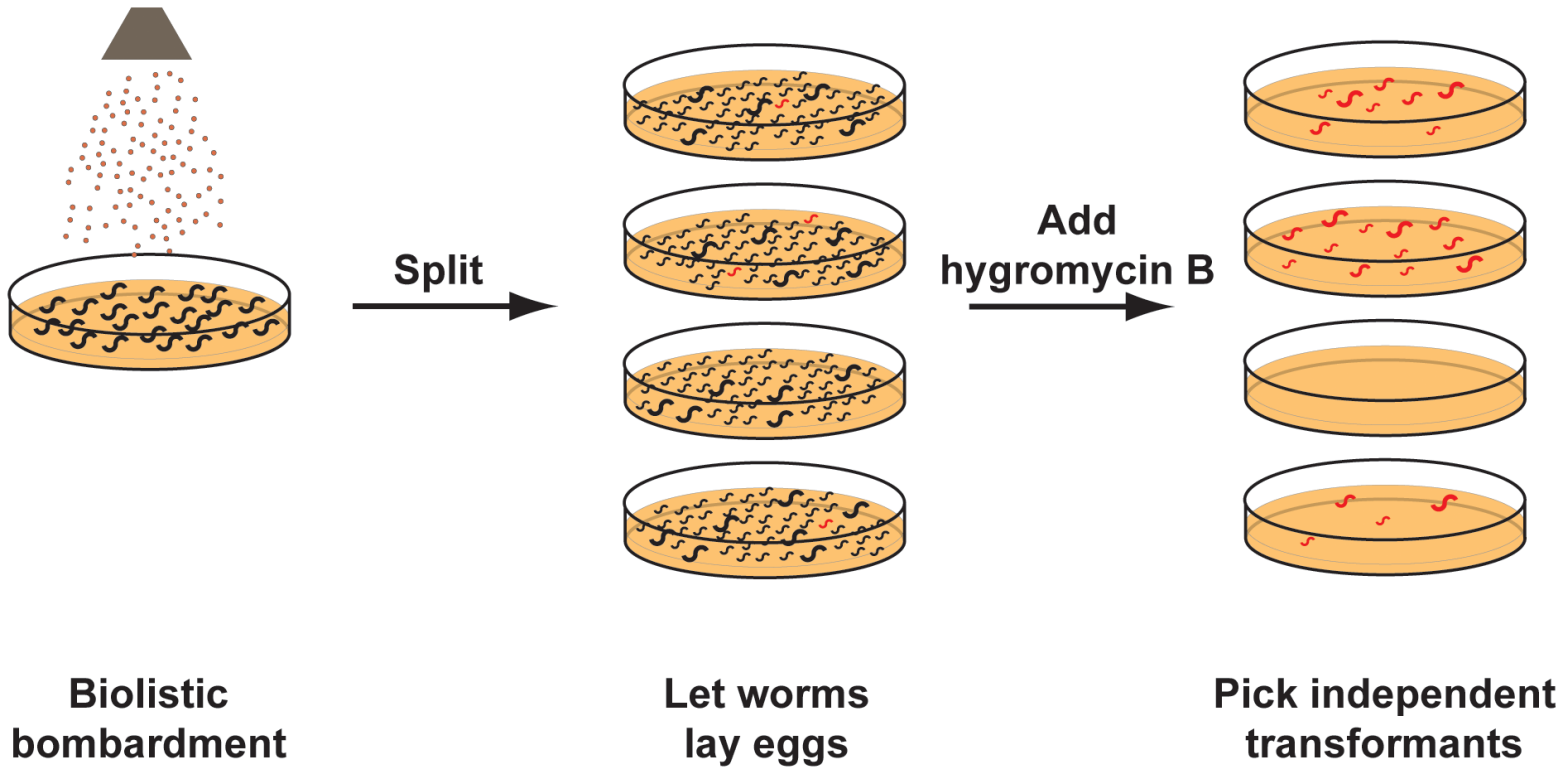
Hygromycin B selection of transgenic *C. elegans* created by biolistic bombardment. Gold beads coated with the DNA mixture of interest, carrying the hygromycin B resistance gene, are bombarded onto worms spread on an agar plate. Bombarded worms are split onto 10–20 selection plates and left to lay eggs for 2–3 days, at which point hygromycin B is added to the plates. After 4–7 days surviving transgenic worms can be picked.

In our optimized bombardment protocol (Figure 2) we grew a population of well-synchronized late L4s/young adults and used about 40 000–90 000 (0.5–1.5 ml settled) worms per bombardment. We found that using 0.3–3 µm gold beads was more cost effective than using 1 µm beads and did not impinge on efficiency (Table S4 in File S1, Table S5 in File S1). Furthermore, switching to 1100 psi rupture disks from the more commonly used 1350 psi disks increased the number of recovered transgenics (Table S4 in File S1, Table S5 in File S1). We demonstrated that DNA linearization prior to bombardment increased the number of transgenics obtained (Table S6 in File S1) [1].

After bombardment, we split the worms onto 10 to 20 plates to allow the subsequent isolation of independent transgenic strains arising from each plate. We let the animals lay eggs for 2–3 days before adding hygromycin B directly onto the surface of the plates. The worms surviving after 4 to 7 days were collected. All worms isolated from this single step selection on hygromycin B were transgenic. A plate of non-transformed worms was routinely included in our selection, and was used to confirm that the selection conditions killed all non-transformed worms.

**Figure 3:**
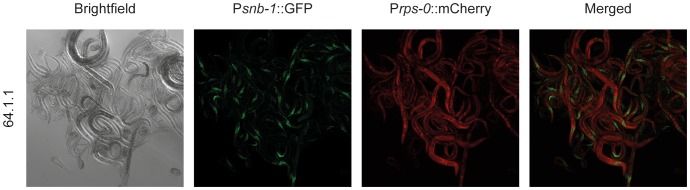
Hygromycin B independent transmission of integrated transgenes. Worms expressing the hygromycin B resistance gene and mCherry from the ubiquitous P*rps-0* promoter and GFP from the pan-neuronal P*snb-1* promoter (P*rps-0*::*HygB,* P*rps-0*::mCherry, P*snb-1*::*GFP*, bombardment 64, strain 64.1.1) were grown as described in Figure S3. Worms were washed off the plate and imaged using a confocal microscope as described in the methods section. The strain was grown and propagated in the absence of hygromycin B.

**Figure 4:**
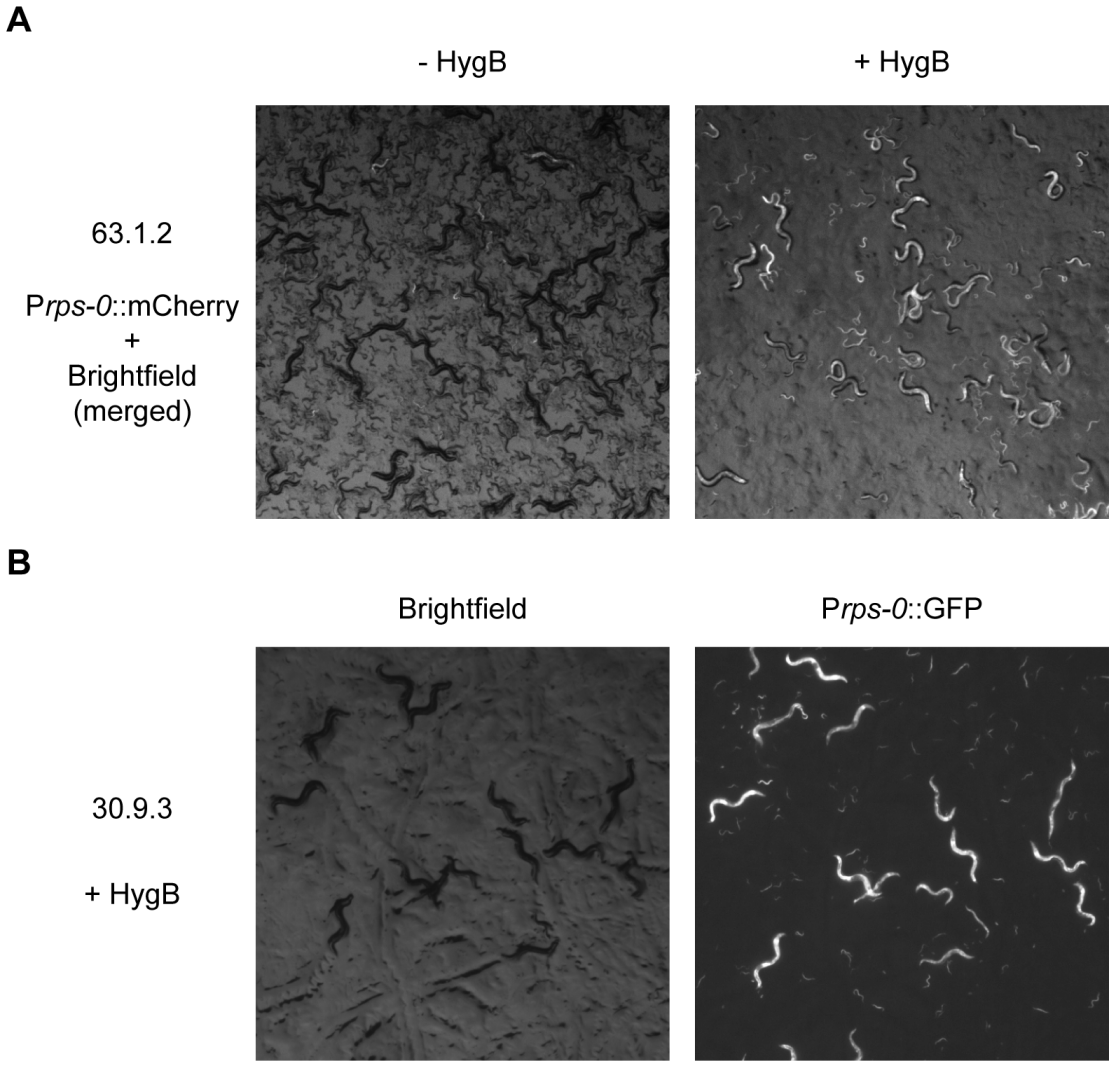
Hygromycin B allows long term maintenance of non-integrated transgenes. a) 3 transgenic worms from a non-integrated line transformed with the hygromycin B resistance and GFP (P*rps-0*::*HygB*::*unc-54,* P*rps-0*::GFP::*unc-54*, bombardment 63, strain 63.1.2) were grown on a seeded plate for 3 generations in the absence and presence of hygromycin B. Worms were imaged as described in the methods section. Exposure time: 700 ms. b) Worms with a non-integrated transgenic array carrying the hygromycin B resistance gene and GFP (P*rps-0*::*HygB*::*unc-54,* P*rps-0*::GFP::*unc-54*, bombardment 30, strain 30.9.3) were propagated for >30 generations in the presence of hygromycin B. Worms were imaged as described in the methods section. Exposure time: 700 ms.

With the optimized approach, based on a large dataset, transgenic strains were obtained from every bombardment (100%) (Table 1). Ten or more independent transmitting transgenic strains were obtained from 84% of bombardments, with an average of 11.6 independent transgenic strains per bombardment (Table 1). The number of independent transformants identified was in many cases limited simply by the number of plates we chose to split the bombardment across and additional experiments demonstrated that the creation of up to 28 independent lines from a single optimized bombardment was possible (Table S6 in File S1). We examined twenty-two bombardments for genomic integration. We found that the large majority of the bombardments (82%) led to an integrated transgenic strain, with an average of 2.4 integrated independent strains per bombardment (Table 1, Figure 3, Figure S3 in File S1, Table S4 in File S1). Since it is likely that some integrated strains generated by biolistic bombardment will carry multiple copies of the transgene, they are likely to give higher expression levels than strains constructed using recently introduced methods for targeted single copy integration [15], [16], which may in some cases be desirable. When working with non-integrated strains, which may be advantageous when high transgene expression levels are desired, we found that we could easily grow and maintain large transgenic cultures at high densities both on agar plates (Figure 4) and in liquid culture (data not shown) in the presence of hygromycin B. When co-bombarding multiple plasmids, we found co-expressing strains in every bombardment. For each bombardment the majority of strains show co-expression. However, we also observed strains that did not express one or more of the bombarded constructs (data not shown).

**Table 1:** Efficiency of biolistic *C. elegans* transgenesis using hygromycin B selection.

Rupture disks	DNA linearized	Bombard-ments	Bombard-ments yielding a transgenic strain (%)	Bombard-ments yielding > = 10 indepen-dent transgenic strains (%)	indepen-dent strains per bombard-ment (average)	Bombard-ments screened for integrants	Bombard-ments yielding an integrated transgenic strain (%)	Bombard-ments yielding > = 2 indepen-dent integrated transgenic strains (%)	indepen-dent integrated strains per bombard-ment (average)
1350	no	24	23 (96%)	5 (21%)	5.75	0	n.d.	n.d.	n.d.
1100	no	11	11 (100%)	4 (36%)	8.09	0	n.d.	n.d.	n.d.
1100	yes	45	45 (100%)	38 (84%)	11.58	22	18 (82%)	15 (68%)	2.4

n.d. – not determined.

## Discussion

We report the direct isolation of transgenic *C. elegans* via biolistic bombardment using hygromycin B, an antibiotic that quantitatively kills worms at low concentration. Unlike previous reports which use combinations of antibiotics and detergent to permeabilize the worms to the antibiotic our approach uses only a single antibiotic and does not require the use of detergents to permeabilize the worms or a subsequent fluorescent reporter screen to isolate transgenic lines.

In contrast to previous methods that use extensive liquid handling, centrifugations and separation steps the method we report uses a single agar based selection, allowing the direct and straightforward isolation of independent lines in a single step. The reduced hands-on time makes the method scalable, and we routinely perform bombardments and selections on ten or more constructs in parallel. Hygromycin B is approximately 15 times less expensive than puromycin, making the use of multiple selection plates, for the straightforward isolation of independent lines, economically feasible for many laboratories.

Crucially, the method provides substantial improvements in the success of bombardments for isolating transmitting strains, the isolation of multiple independent strains, and the isolation of integrated strains. While previous methods give transmitting strains in up to 80% of transformations, the method we developed yielded transmitting strains in 100% of bombardments. Moreover, our method allows us to routinely isolate multiple independent transmitting strains, indeed 84% of bombardments yielded greater than ten independent transmitting strains with bombardments yielding up to 28 independent transmitting strains. While previous methods yield an integrated strain from up to 70% of bombardments (Ben Lehner, personal communication) we find integrated strains in 82% of bombardments, and we find greater than two independent integrated strains in 68% of bombardments. Using our approach a single bombardment suffices to obtain both an integrated transgenic strain and a variety of extrachromosomal transgenic strains with a range of transgene copy number and transgene expression levels that can be stably maintained.

While we have focused on characterizing hygromycin B based selections for bombardment based transgenesis, the selection will be useful for marking the introduction or deletion of DNA into *C. elegans* by other methods. Indeed, the hygromycin B cassette we have reported here can also be used to select targeted knock-ins generated using a CRISPR-Cas9 system in *C. elegans* [17].

## Supporting Information

File S1 **Contains: Figure S1.** Survival of *C. elegans* on hygromycin B. Synchronized L1 larvae were added to seeded 6 cm NGM plates and immediately treated with the indicated hygromycin B concentrations. Plates were imaged 40 hours after treatment. a) At 300 worms per plate no treated worms reached the L4 stage even at the lowest hygromycin B concentration. b) At 3000 worms per plate some animals reached the L4 stage at the lowest antibiotic concentration, whereas at higher concentrations no animals reached the L4 stage. **Figure S2.** Gateway-based vectors constructed for the selection of hygromycin B resistant transgenics. a) Basic hygromycin B resistance vectors. b) New optimized hygromycin B resistance vectors. *unc-54* 3′UTR –3′ untranscribed downstream region of *unc-54*. *Amp* – ampicillin resistance gene. ori – origin of replication. attR4 & attR3– Gateway recombination sites. *ccdB* – toxicity gene. CAT - chloramphenicol acetyltransferase gene. MCS1 - *KasI*/*NarI*/*SfoI*, *SpeI* unique restriction sites. MCS2– *KpnI*, *NheI*, *AvrII*, *AscI* unique restriction sites. P*rps-0*_short – shorter upstream region (0.8 kb) of *rps-0*. *HygR* CeOPT – *C. elegans* optimized (codon optimization, introns) hygromycin B phosphotransferase gene. *gpd-2/gpd-3* outron – CEOPX036 operon intergenic region (outron) between *gpd-2* and *gpd-3* genes. GFP – green fluorescent protein gene (optimized for *C. elegans*). mCherry – mCherry fluorescent protein gene (optimized for *C. elegans*). **Figure S3.** Hygromycin B independent transmission of integrated transgenes. Worms were judged to contain integrated transgenes using previously reported criteria: transmission frequency of 100%, no mosaicism in any worm [10]. An individual animal from an integrated transgenic strain was transferred to a single plate and a population grown until food was exhausted, at which time progeny were twice more transferred to fresh plates by chunking. The hygromycin B resistance gene and GFP were expressed under the control of the ubiquitous P*rps-0* promoter (P*rps-0*::*HygR*::*gpd-2/gpd-3*::GFP::*unc-*54, bombardment 61, strains 61.4.3, 61.10.2; P*rps-0_*short::*HygR,* P*rps-0*::GFP::*unc-*54, bombardment 65, strain 65.2.1). Worms were imaged as described in the methods section. Exposure time: 700 ms. All of the strains were grown and propagated in the absence of hygromycin B. **Table S1.** Survival of *C. elegans* on hygromycin B. 300 (low density) or 3000 (high density) synchronized L1 larvae were plated on 6 cm plates and immediately treated with hygromycin B. The plates were then scored for the number of of L4 larvae (after ∼40 hours) and adult animals (after ∼60 and ∼90 hours). The experiment was repeated in triplicate. **Table S2.** Primers used for vector construction. **Table S3.** List of plasmids generated. **Table S4.** Detailed summary of all bombardments performed. **Table S5.** Optimization of bombardment conditions. The upper table shows the effect of rupture discs on transformation efficiency (rupture discs define the force at which gold beads are shot onto the worms). The lower table shows the effect of bead size in combination with different types of rupture discs. **Table S6.** Influence of DNA linearization on bombardment efficiency. To test the importance of DNA linearization on transformation efficiency, a single DNA mixture was prepared and then further processed in 3 different ways (non-linearized, linearized but not purified before coating, linearized and purified before coating). Purification was performed using a PCR purification kit (Qiagen). **Supplementary Note.**
(DOC)
