# Supplementary material for: Efficient and Rapid C. elegans Transgenesis by Bombardment and Hygromycin B Selection
Source: PLoS One. 2013 Oct 9;8(10):e76019. doi: 10.1371/journal.pone.0076019 (PMC3794024; doi:10.1371/journal.pone.0076019)
Supplement: File S1 — Contains: Figure S1. Survival of C. elegans on hygromycin B. Synchronized L1 larvae were added to seeded 6 cm NGM plates and immediately treated with the indicated hygromycin B concentrations. Plates were imaged 40 hours after treatment. a) At 300 worms per plate no treated worms reached the L4 stage even at the lowest hygromycin B concentration. b) At 3000 worms per plate some animals reached the L4 stage at the lowest antibiotic concentration, whereas at higher concentrations no animals reached the L4 stage. Figure S2. Gateway-based vectors constructed for the selection of hygromycin B resistant transgenics. a) Basic hygromycin B resistance vectors. b) New optimized hygromycin B resistance vectors. unc-54 3′UTR –3′ untranscribed downstream region of unc-54. Amp – ampicillin resistance gene. ori – origin of replication. attR4 & attR3– Gateway recombination sites. ccdB – toxicity gene. CAT - chloramphenicol acetyltransferase gene. MCS1 - KasI/NarI/SfoI, SpeI unique restriction sites. MCS2– KpnI, NheI, AvrII, AscI unique restriction sites. Prps-0_short – shorter upstream region (0.8 kb) of rps-0. HygR CeOPT – C. elegans optimized (codon optimization, introns) hygromycin B phosphotransferase gene. gpd-2/gpd-3 outron – CEOPX036 operon intergenic region (outron) between gpd-2 and gpd-3 genes. GFP – green fluorescent protein gene (optimized for C. elegans). mCherry – mCherry fluorescent protein gene (optimized for C. elegans). Figure S3. Hygromycin B independent transmission of integrated transgenes. Worms were judged to contain integrated transgenes using previously reported criteria: transmission frequency of 100%, no mosaicism in any worm [10]. An individual animal from an integrated transgenic strain was transferred to a single plate and a population grown until food was exhausted, at which time progeny were twice more transferred to fresh plates by chunking. The hygromycin B resistance gene and GFP were expressed under the control of the ubiquitous Prps-0 promoter ( [file pone.0076019.s001.doc]

**Figure S1**

**
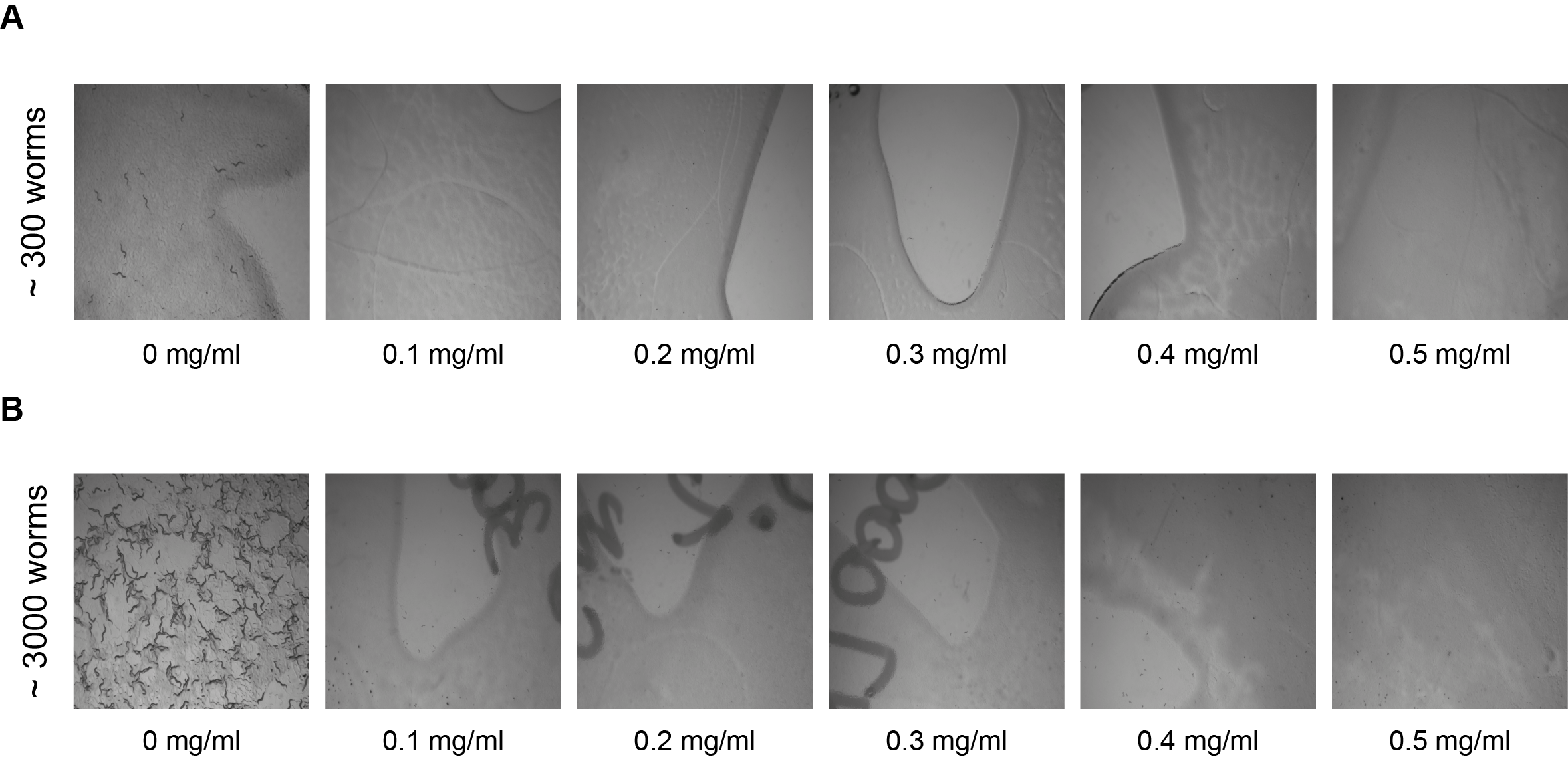
**

**Figure S2**

**
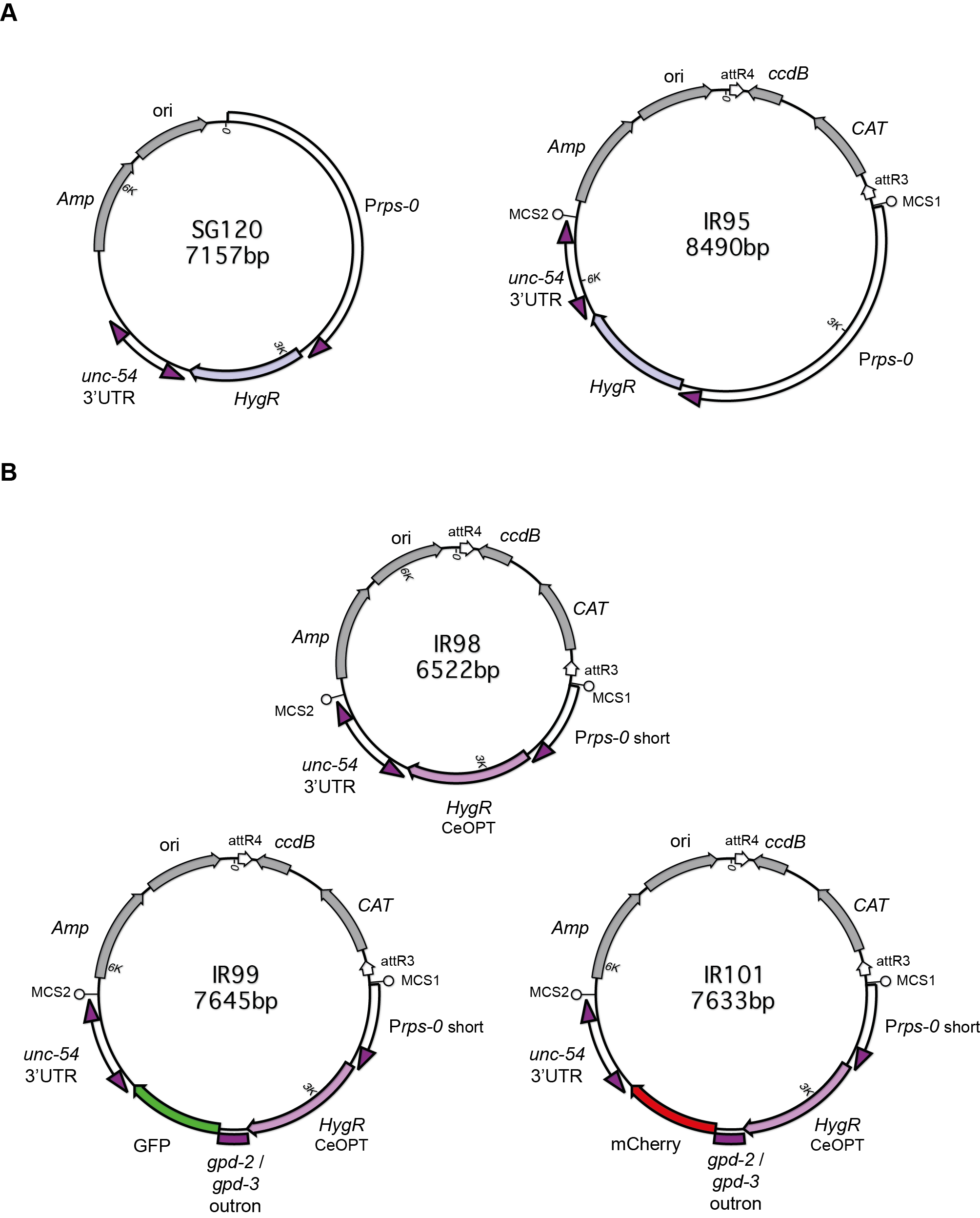
**

**Figure S3**

**
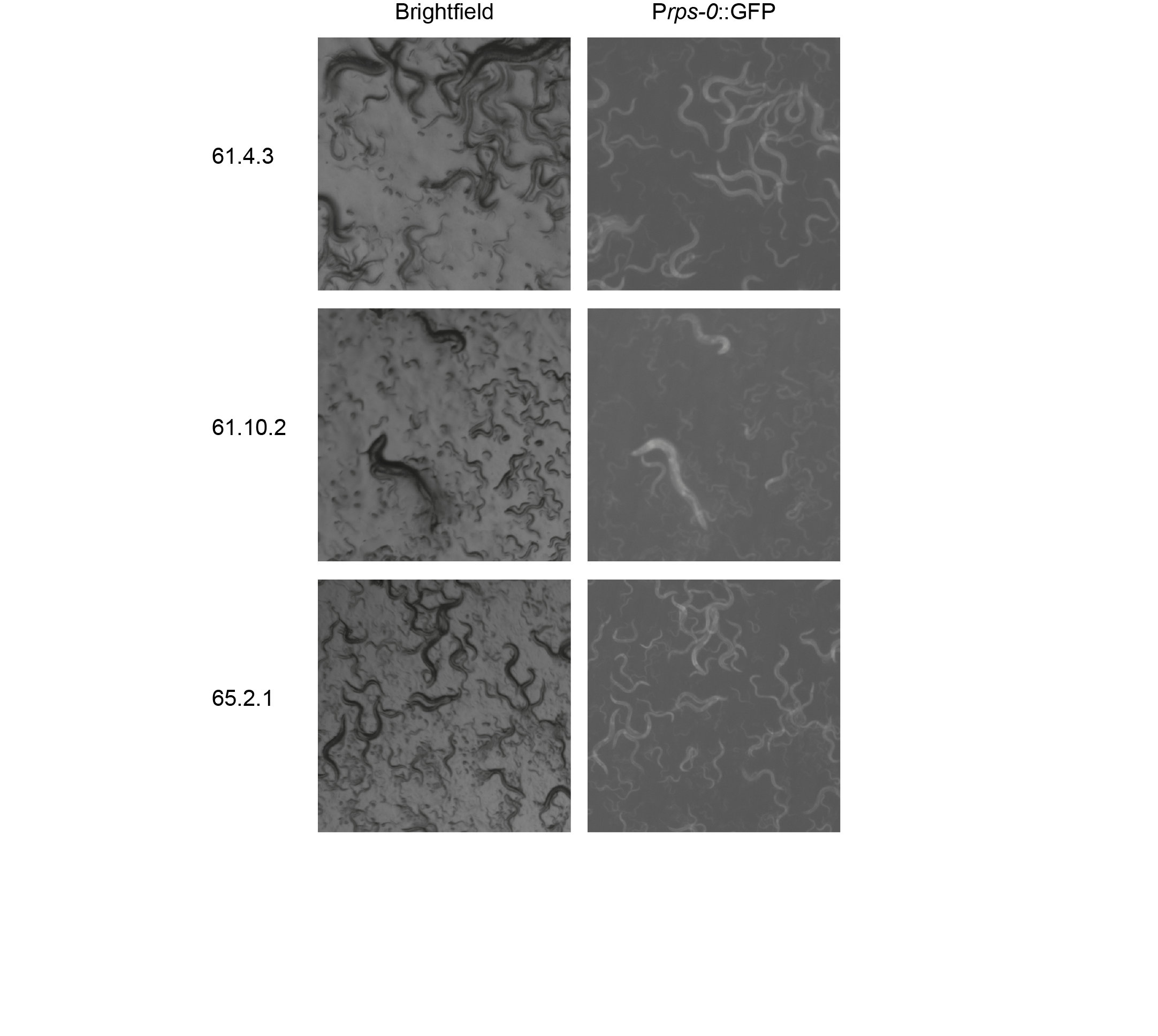
**

**Table S1.**

| 300L1 / 6 cm plate | | | | |
| --- | --- | --- | --- | --- |
| **Concentration (mg ml^-1^)** | **L4 ( after ~ 40 hours)** | **Adults ( after ~ 60 hours)** | **Adults ( after ~ 90 hours)** | **Killing efficiency (%)** |
| 0.1 | 0 | 0 | 0 | 100 |
| 0.2 | 0 | 0 | 0 | 100 |
| 0.3 | 0 | 0 | 0 | 100 |
| 0.4 | 0 | 0 | 0 | 100 |
| 0.5 | 0 | 0 | 0 | 100 |

| 3000L1 / 6 cm plate | | | | |
| --- | --- | --- | --- | --- |
| **Concentration (mg ml^-1^)** | **L4 ( after ~ 40 hours)** | **Adults ( after ~ 60 hours)** | **Adults ( after ~ 90 hours)** | **Killing efficiency (%)** |
| 0.1 | 1.67 | 3 | 5.34 | 99.82 |
| 0.2 | 0 | 0 | 0 | 100 |
| 0.3 | 0 | 0 | 0 | 100 |
| 0.4 | 0 | 0 | 0 | 100 |
| 0.5 | 0 | 0 | 0 | 100 |

**Table S2.**

| **PRIMER** | **SEQUENCE** | **REGION TO AMPLIFY** |
| --- | --- | --- |
| WP21_attB4 – Prps-0_long F (pos1) | GGGGACAACTTTGTATAGAAAAGTTGTTGGGTGAAAGGAACGACAC | P*rps-0*_long |
| WP43_attB1– Prps-0_long F (pos2) | GGGGACAAGTTTGTACAAAAAAGCAGGCTTTGGGTGAAAGGAACGACAC | P*rps-0*_long |
| WP61_attB4 – Prps-0_short F | GGGGACAACTTTGTATAGAAAAGTTGATGAAGATGAACGCAAGGAG | P*rps-0*_short |
| WP62_attB1– Prps-0_short F | GGGGACAAGTTTGTACAAAAAAGCAGGCTATGAAGATGAACGCAAGGAG | P*rps-0*_short |
| WP64_Prps-0 R | ATTACCTTAAAATTCAAAAATTAATTTC | P*rps-0_*long / P*rps-0*_short |
| WP65_Prps-0 – Hyg_nonOPT F | AATTTTTGAATTTTAAGGTAATATGAAAAAGCCTGAACTCAC | *HygR* |
| WP66_Prps-0 – Hyg_CeOPT F | AATTTTTGAATTTTAAGGTAATATGAAAAAACCCGAGTTGAC | *HygR C.elegans* optimized |
| WP68_Unc-54 5’overlap – Hyg_nonOPT R | GACCGGCGCTCAGTTGGAATTCTATTCCTTTGCCCTCGGAC | *HygR* |
| WP69_Unc-54 5’overlap – Hyg_CeOPT R | GACCGGCGCTCAGTTGGAATTTTACTCCTGAAAATTTAAATATG | *HygR C.elegans* optimized |
| WP127_gpd-2/3_operon_intragen 5’overlap – Hyg_nonOPT R | CTAGGTGAAAGTAGGATGAGACAGCCTATTCCTTTGCCCTCGGAC | *HygR* |
| WP76_gpd-2/3_operon_intragen 5’overlap – Hyg_CeOPT R | CTAGGTGAAAGTAGGATGAGACAGCTTACTCCTGAAAATTTAAATATG | *HygR C.elegans* optimized |
| WP23_unc-54 5’ overlap F | AATTCCAACTGAGCGCCGGTC | *unc-54* 3’UTR |
| WP39_attB1– unc-54 3’ overlap R (pos1) | GGGGACTGCTTTTTTGTACAAACTTGCAAAGTTGAAACAGTTATGTTTG | *unc-54* 3’UTR |
| WP48_attB2 – unc-54 3’ overlap R (pos2) | GGGGACCACTTTGTACAAGAAAGCTGGGTAAAGTTGAAACAGTTATGTTTG | *unc-54* 3’UTR |
| WP77_gpd-2/3_operon_intragen 5’overlap F | GCTGTCTCATCCTACTTTCACCTAG | *gpd-2/gpd-3* outron |
| WP80_GFP - gpd-2/3_operon_intragen R | GAGCTCCTCTCCCTTGGACATTTTTTCTACCGGTACAGCAGTTTC | *gpd-2/gpd-3* outron |
| WP82_mCherry - gpd-2/3_operon_intragen R | CTTCTTCACCCTTTGAGACCATTTTTTCTACCGGTACAGCAGTTTC | *gpd-2/gpd-3* outron |
| WP44_GFP F | ATGTCCAAGGGAGAGGAGCTC | GFP *C.elegans* optimized |
| WP78_mCherry F | ATGGTCTCAAAGGGTGAAGAAG | mCherry *C.elegans* optimized |
| WP84_Unc-54 5’overlap – GFP R | GACCGGCGCTCAGTTGGAATTTCCTCCCTTGTAGAGCTCGTCCATTC | GFP *C.elegans* optimized |
| WP85_Unc-54 5’overlap – mCherry R | GACCGGCGCTCAGTTGGAATTCTTATACAATTCATCCATGCCACCTG | mCherry *C.elegans* optimized |
| WP189_AscI_AvrII_ NheI_KpnI F pDEST_amp_F 1 | taaaGGCGCGCCaaaaCCTAGGaaaaGCTAGCaaaaGGTACCTGGTTTCTTAGACGTCAGGTGGCACTTTTCGGGGAAATG | pDEST vector backbone |
| WP190_SpeI_KasI/NarI/SfoI pDEST_amp_R 1 | taaaACTAGTaaaaGGCGCCAGGCGATTAAGTTGGGTAACGCCAGGGTTTTCCCAGTCAC | pDEST vector backbone |
| WP191_pINS F 1 (pDEST_KasI_SpeI) Prps-0_long F | ACCCTGGCGTTACCCAACTTAATCGCCTGGCGCCttttACTAGTtttaGGGTGAAAGGAACGACACTATGTACATCAACCAG | Any construct starting with P*rps-0*_long |
| WP193_pINS F 1 (pDEST_KasI_SpeI) Prps-0_short F | ACCCTGGCGTTACCCAACTTAATCGCCTGGCGCCttttACTAGTtttaATGAAGATGAACGCAAGGAGCCTGATGCAGCGACTC | Any construct starting with P*rps-0*_short |
| WP195_pINS R 1 (pDEST_MCS) unc54 R | TAAGAAACCAGGTACCttttGCTAGCttttCCTAGGttttGGCGCGCCtttaAGTTGAAACAGTTATGTTTGGTATATTGGGAATG | Any construct ending with *unc-54* 3’UTR |

**Table S3.**

| PLASMID NAME | GATEWAY INSERT | BACKBONE INSERT | PARENTAL GATEWAY PLASMID |
| --- | --- | --- | --- |
| SG120 | P*rps-0::HygR*::*unc-54*_3'UTR | - | pDEST R4-R3 |
| IR83 | P*rps-0_*sh::*HygR*_CeOPT::*gpd-2/gpd-3*::*GFP*::*unc-54*_3'UTR | - | pDONR 221 |
| IR84 | P*rps-0::HygR*::*unc-54*_3'UTR | - | pDONR P4-P1R |
| IR85 | P*rps-0*_sh*::HygR*::*unc-54*_3'UTR | - | pDONR P4-P1R |
| IR86 | P*rps-0::HygR*_CeOPT::*unc-54*_3'UTR | - | pDONR 221 |
| IR87 | P*rps-0*_sh*::HygR*_CeOPT::*unc-54*_3'UTR | - | pDONR 221 |
| IR88 | P*rps-0*::*HygR*_CeOPT::*gpd-2/gpd-3*::*mCherry*::*unc-54*_3'UTR | - | pDONR 221 |
| IR89 | P*rps-0*::*HygR*::*gpd-2/gpd-3*::*GFP*::*unc-54*_3'UTR | - | pDONR 221 |
| IR90 | P*rps-0*::*HygR*::*gpd-2/gpd-3*::*mCherry*::*unc-54*_3'UTR | - | pDONR 221 |
| IR95 | - | P*rps-0::HygR*::*unc-54*_3'UTR | pDEST R4-R3 |
| IR96 | - | P*rps-0*_sh*::HygR*::*unc-54*_3'UTR | pDEST R4-R3 |
| IR97 | - | P*rps-0::HygR*_CeOPT::*unc-54*_3'UTR | pDEST R4-R3 |
| IR98 | - | P*rps-0*_sh*::HygR*_CeOPT::*unc-54*_3'UTR | pDEST R4-R3 |
| IR99 | - | P*rps-0_*sh::*HygR*_CeOPT::*gpd-2/gpd-3*::*GFP*::*unc-54*_3'UTR | pDEST R4-R3 |
| IR100 | - | P*rps-0*::*HygR*_CeOPT::*gpd-2/gpd-3*::*mCherry*::*unc-54*_3'UTR | pDEST R4-R3 |
| IR101 | - | P*rps-0*_sh::*HygR*_CeOPT::*gpd-2/gpd-3*::*mCherry*::*unc-54*_3'UTR | pDEST R4-R3 |
| IR102 | - | P*rps-0*::*HygR*::*gpd-2/gpd-3*::*GFP*::*unc-54*_3'UTR | pDEST R4-R3 |
| IR103 | - | P*rps-0*_sh::*HygR*::*gpd-2/gpd-3*::*GFP*::*unc-54*_3'UTR | pDEST R4-R3 |
| IR104 | - | P*rps-0*::*HygR*::*gpd-2/gpd-3*::*mCherry*::*unc-54*_3'UTR | pDEST R4-R3 |
| IR105 | - | P*rps-0*_sh::*HygR*::*gpd-2/gpd-3*::*mCherry*::*unc-54*_3'UTR | pDEST R4-R3 |
| IR106 | P*snb-1*::*GFP*::*let-858*_3'UTR | P*rps-0::HygR*::*unc-54*_3'UTR | IR95 (pDEST R4-R3) |
| IR107 | P*snb-1*::*GFP*::*let-858*_3'UTR | P*rps-0::HygR*::*unc-54*_3'UTR | IR95 (pDEST R4-R3) |
| IR108 | P*snb-1*::*GFP*::*let-858*_3'UTR | P*rps-0::HygR*_CeOPT::*unc-54*_3'UTR | IR97 (pDEST R4-R3) |
| IR109 | P*snb-1*::*mCherry*::*let-858*_3'UTR | P*rps-0*::*HygR*::*gpd-2/gpd-3*::*GFP*::*unc-54*_3'UTR | IR102 (pDEST R4-R3) |
| IR110 | P*snb-1*::*mCherry*::*let-858*_3'UTR | P*rps-0*::*HygR*::*gpd-2/gpd-3*::*GFP*::*unc-54*_3'UTR | IR102 (pDEST R4-R3) |

**Table S4.**

| **N (bomb)** | **STRAIN** | **DNA MIXTURE** | **RUPTURE**  **DISKS (psi)** | **DNA**  **LINEARIZATION** | **V worms (ml)** | **N (selection**  **Plates)** | **N (min. independent**  **transmitting strains)** | **N (min. independent**  **integrated strains)** |
| --- | --- | --- | --- | --- | --- | --- | --- | --- |
| 1 | *smg-2* | 1 μg IR23* , 3 μg SG88*, 3 μg SG132, 3 μg SG120^Δ^ | 1350 | no | 1 | 14 | 10 | n.d. |
| 2 | *smg-2* | 1 μg IR23* , 13 μg SG132, 6 μg SG120^Δ^ | 1350 | no | 1 | 14 | 8 | n.d. |
| 3 | *smg-2* | 1 μg IR23* , 3 μg SG132, 6 μg SG120^Δ^ | 1350 | no | 1 | 14 | 2 | n.d. |
| 4 | *smg-2* | 1 μg IR25* , 3 μg SG88*, 3 μg SG131, 3 μg SG120^Δ^ | 1350 | no | 1 | 14 | 7 | n.d. |
| 5 | *smg-2* | 1 μg IR27* , 3 μg SG88*, 3 μg SG134, 3 μg SG120^Δ^ | 1350 | no | 1 | 14 | 3 | n.d. |
| 6 | *smg-2* | 1 μg IR28* , 3 μg SG88*, 3 μg SG163, 3 μg SG120^Δ^ | 1350 | no | 1 | 14 | 0 | n.d. |
| 7 | *smg-2* | 1 μg IR29* , 3 μg SG88*, 3 μg SG164, 3 μg SG120^Δ^ | 1350 | no | 1 | 14 | 3 | n.d. |
| 8 | *smg-2* | 1 μg IR30* , 3 μg SG88*, 3 μg SG166, 3 μg SG120^Δ^ | 1350 | no | 1 | 14 | 3 | n.d. |
| 9 | *smg-2* | 1 μg IR31* , 3 μg SG88*, 3 μg SG167, 3 μg SG120^Δ^ | 1350 | no | 1 | 14 | 7 | n.d. |
| 10 | *smg-2* | 1 μg IR59 , 3 μg SG88, 3 μg IR9, 3 μg SG120^Δ^ | 1350 | no | 1 | 14 | 8 | n.d. |
| 11 | *smg-2* | 1 μg IR57*, 3 μg SG88*, 3 μg SG132, 3 μg SG120^Δ^ | 1350 | no | 1 | 14 | 13 | n.d. |
| 12 | *smg-2* | 1 μg IR57*, 9 μg SG120^Δ^ | 1350 | no | 1 | 14 | 12 | n.d. |
| 13 | *smg-2* | 1 μg IR57*, 3 μg SG132, 6 μg SG120^Δ^ | 1350 | no | 1 | 14 | 2 | n.d. |
| 14 | *smg-2* | 1 μg IR58*, 3 μg SG88*, 3 μg SG132, 3 μg SG120^Δ^ | 1350 | no | 1 | 14 | 2 | n.d. |
| 15 | *smg-2* | 1 μg IR58*, 9 μg SG120^Δ^ | 1350 | no | 1 | 14 | 10 | n.d. |
| 16 | *smg-2* | 1 μg IR58*, 3 μg SG132, 6 μg SG120^Δ^ | 1350 | no | 1 | 14 | 8 | n.d. |
| 17 | *smg-2* | 1 μg IR65*, 9 μg SG120^Δ^ | 1350 | no | 1 | 14 | 5 | n.d. |
| 18 | *smg-2* | 1 μg IR65*, 3 μg SG88*, 3 μg SG132, 3 μg SG120^Δ^ | 1350 | no | 1 | 14 | 10 | n.d. |
| 19 | *smg-2* | 1 μg IR67*, 9 μg SG120^Δ^ | 1350 | no | 1 | 14 | 3 | n.d. |
| 20 | *smg-2* | 1 μg IR67*, 3 μg SG88*, 3 μg SG132, 3 μg SG120^Δ^ | 1350 | no | 1 | 14 | 9 | n.d. |
| 21 | *smg-2* | 1 μg IR60*, 3 μg SG88*, 3 μg SG132, 3 μg SG120^Δ^ | 1350 | no | 0.5 | 14 | 3 | n.d. |
| 22 | *smg-2* | 1 μg IR60*, 9 μg SG120^Δ^ | 1350 | no | 0.5 | 14 | 4 | n.d. |
| 23 | *smg-2* | 1 μg IR61*, 3 μg SG88*, 3 μg SG132, 3 μg SG120^Δ^ | 1350 | no | 0.5 | 14 | 3 | n.d. |
| 24 | *smg-2* | 1 μg IR61*, 9 μg SG120^Δ^ | 1350 | no | 0.5 | 14 | 3 | n.d. |
| 25 | *smg-2* | 1 μg IR62*, 3 μg SG88*, 3 μg SG132, 3 μg SG120^Δ^ | 1100 | no | 0.5 | 14 | 7 | n.d. |
| 26 | *smg-2* | 1 μg IR62*, 9 μg SG120^Δ^ | 1100 | no | 0.5 | 14 | 10 | n.d. |
| 27 | *smg-2* | 1 μg IR63*, 3 μg SG88*, 3 μg SG132, 3 μg SG120^Δ^ | 1100 | no | 0.5 | 14 | 11 | n.d. |
| 28 | *smg-2* | 1 μg IR63*, 9 μg SG120^Δ^ | 1100 | no | 0.5 | 14 | 6 | n.d. |
| 29 | *smg-2* | 1 μg SG250*, 3 μg SG132, 6 μg SG120^Δ^ | 1100 | no | 0.6 | 16 | 3 | n.d. |
| 30 | *smg-2* | 1 μg SG245*, 3 μg SG88*, 3 μg SG132, 3 μg SG120^Δ^ | 1100 | no | 0.6 | 16 | 13 | n.d. |
| 31 | *smg-2* | 1.5 μg SG82, 3 μg SG88*, 3 μg IR9, 2.5 μg SG120^Δ^ | 1100 | no | 0.6 | 16 | 7 | n.d. |
| 32 | *smg-2* | 1 μg SG251*, 3 μg SG132, 6 μg SG120^Δ^ | 1100 | no | 1.2 | 16 | 5 | n.d. |
| 33 | *smg-2* | 1 μg SG247*, 3 μg SG132, 6 μg SG120^Δ^ | 1100 | no | 1.2 | 16 | 8 | n.d. |
| 34 | *smg-2* | 1 μg SG249*, 3 μg SG132, 6 μg SG120^Δ^ | 1100 | no | 1.2 | 16 | 9 | n.d. |
| 35 | *smg-2* | 1 μg SG238*, 3 μg SG132, 6 μg SG120^Δ^ | 1100 | no | 1.2 | 30 | 10 | n.d. |
| 36 | *smg-2* | 1 μg SG238*, 3 μg SG132, 6 μg SG120^Δ^ | 1100 | SfoI, NotI (DNA not purified after, directly coated) | 1.2 | 30 | 23 | n.d. |
| 37 | *smg-2* | 1 μg SG238*, 3 μg SG132, 6 μg SG120^Δ^ | 1100 | SfoI, NotI (DNA purified after, then coated) | 1.2 | 30 | 28 | n.d. |
| 38 | *smg-2* | 1 μg SG233*, 3 μg SG88*, 3 μg SG132, 3 μg SG120^Δ^ | 1100 | SfoI, NotI | 1 | 20 | 10 | n.d. |
| 39 | *smg-2* | 1 μg SG234*, 3 μg SG88*, 3 μg SG132, 3 μg SG120^Δ^ | 1100 | SfoI, NotI | 1 | 20 | 15 | n.d. |
| 40 | *smg-2* | 1 μg SG235*, 3 μg SG88*, 3 μg SG132, 3 μg SG120^Δ^ | 1100 | SfoI, NotI | 1 | 20 | 14 | n.d. |
| 41 | *smg-2* | 1 μg SG237*, 3 μg SG88*, 3 μg SG132, 3 μg SG120^Δ^ | 1100 | SfoI, NotI | 1 | 20 | 14 | n.d. |
| 42 | *smg-2* | 1 μg SG239*, 3 μg SG88*, 3 μg SG132, 3 μg SG120^Δ^ | 1100 | SfoI, NotI | 1 | 20 | 15 | n.d. |
| 43 | *smg-2* | 1 μg SG240*, 3 μg SG88*, 3 μg SG132, 3 μg SG120^Δ^ | 1100 | SfoI, NotI | 1 | 20 | 14 | n.d. |
| 44 | *smg-2* | 1 μg SG242*, 3 μg SG88*, 3 μg SG132, 3 μg SG120^Δ^ | 1100 | SfoI, NotI | 1 | 20 | 14 | n.d. |
| 45 | *smg-2* | 1 μg SG244*, 3 μg SG88*, 3 μg SG132, 3 μg SG120^Δ^ | 1100 | SfoI, NotI | 1 | 20 | 13 | n.d. |
| 46 | N2 | 1 μg IR76*, 3 μg SG132, 6 μg SG120^Δ^ | 1100 | SfoI, NotI | 0.8 | 14 | 12 | n.d. |
| 47 | N2 | 1 μg IR77*, 3 μg SG132, 6 μg SG120^Δ^ | 1100 | SfoI, NotI | 0.8 | 14 | 12 | n.d. |
| 48 | N2 | 1 μg IR78* ^Δ^, 9 μg SG132 | 1100 | SfoI, NotI | 0.8 | 14 | 1 | n.d. |
| 49 | N2 | 1 μg IR79* ^Δ^, 9 μg SG132 | 1100 | SfoI, NotI | 0.8 | 14 | 2 | n.d. |
| 50 | N2 | 10 μg IR78* ^Δ^ | 1100 | SfoI | 0.8 | 12 | 7 | n.d. |
| 51 | N2 | 10 μg IR79* ^Δ^ | 1100 | SfoI | 0.8 | 12 | 10 | n.d. |
| 52 | *smg-2* | 3 μg IR97^Δ^, 7 μg SG343* | 1100 | AscI, PciI | 0.5 | 10 | 10 | n.d. |
| 53 | *smg-2* | 3 μg IR97^Δ^, 7 μg SG343* | 1100 | AscI, PciI | 0.5 | 10 | 10 | n.d. |
| 54 | *smg-2* | 3 μg IR97^Δ^, 7 μg SG343* | 1100 | AscI, PciI | 0.5 | 10 | 8 | n.d. |
| 55 | N2 | 10 μg IR89* ^Δ^ | 900 | PciI | 0.8 | 12 | 8 | n.d. |
| 56 | N2 | 10 μg IR89* ^Δ^ | 1100 | PciI | 0.8 | 12 | 10 | 1 |
| 57 | N2 | 10 μg IR95-2* ^Δ^ | 1100 | SfoI | 0.8 | 12 | 8 | n.d. |
| 58 | N2 | 10 μg IR96* ^Δ^ | 1100 | SfoI | 0.8 | 12 | 8 | n.d. |
| 59 | N2 | 10 μg IR98* ^Δ^ | 1100 | SfoI | 1 | 12 | 12 | n.d. |
| 60 | N2 | 10 μg IR102* ^Δ^ | 1100 | SfoI | 1 | 12 | 12 | 3 |
| 61 | N2 | 10 μg IR102* ^Δ^ | 1100 | SfoI | 1 | 12 | 12 | 6 |
| 62 | N2 | 10 μg IR83* ^Δ^ | 1100 | PciI | 1 | 10 | 10 | 1 |
| 63 | N2 | 7 μg IR106* ^Δ^, 3 μg SG343* | 1100 | KasI | 1 | 10 | 10 | 2 |
| 64 | N2 | 7 μg IR107* ^Δ^, 3 μg SG343* | 1100 | KasI | 1 | 10 | 10 | 2 |
| 65 | N2 | 7 μg IR96 ^Δ^, 3 μg SG343* | 1100 | KasI | 1 | 10 | 10 | 4 |
| 66 | N2 | 7 μg IR108* ^Δ^, 3 μg SG343* | 1100 | KasI | 1 | 10 | 10 | 0 |
| 67 | N2 | 7 μg IR98 ^Δ^, 3 μg SG344* | 1100 | KasI | 1 | 19 | 19 | 1 |
| 68 | N2 | 7 μg IR98 ^Δ^, 3 μg SG344* | 1100 | SfoI | 1 | 19 | 18 | 0 |
| 69 | N2 | 6 μg IR109* ^Δ^, 4 μg pDEST (Gateway) | 1100 | KasI | 1 | 10 | 10 | 3 |
| 70 | N2 | 6 μg IR110* ^Δ^, 4 μg pDEST (Gateway) | 1100 | KasI | 1 | 10 | 10 | 5 |
| 71 | N2 | 10 μg IR99* ^Δ^ | 1100 | PciI | 1.5 | 12 | 10 | 2 |
| 72 | N2 | 10 μg IR100* ^Δ^ | 1100 | PciI | 1.5 | 12 | 11 | 2 |
| 73 | N2 | 10 μg IR101* ^Δ^ | 1100 | PciI | 1.5 | 12 | 12 | 2 |
| 74 | N2 | 10 μg IR103* ^Δ^ | 1100 | PciI | 1.5 | 12 | 11 | 0 |
| 75 | N2 | 10 μg IR104* ^Δ^ | 1100 | PciI | 1.5 | 12 | 12 | 4 |
| 76 | N2 | 10 μg IR105* ^Δ^ | 1100 | PciI | 1.5 | 12 | 12 | 5 |
| 77 | N2 | 5 μg IR84^Δ^, 5 μg SG343* | 1100 | PciI | 1 | 12 | 10 | 5 |
| 78 | N2 | 5 μg IR85^Δ^, 5 μg SG343* | 1100 | PciI | 1 | 12 | 12 | 0 |
| 79 | N2 | 5 μg IR86^Δ^, 5 μg SG343* | 1100 | PciI | 1 | 12 | 10 | 2 |
| 80 | N2 | 5 μg IR87^Δ^, 5 μg SG343* | 1100 | PciI | 1 | 12 | 12 | 3 |

* plasmids carrying a fluorescent marker (GFP or mCherry)

^Δ^ plasmids carrying hygromycin B resistance

n.d. – not determined

**Table S5.**

| Bombardment Set 1 | | | | |  |
| --- | --- | --- | --- | --- | --- |
| **DNA MIXTURE** | **RUPTURE**  **DISKS (psi)** | **DNA**  **LINEARIZATION** | **V worms (ml)** | **N (selection**  **plates)** | **N (min. independent**  **transmitting strains)** |
| 1 μg IR60, 3 μg SG88, 3 μg SG132, 3 μg SG120 | 1350 | no | 0.5 | 14 | 3 |
| 1 μg IR60, 9 μg SG120 | 1350 | no | 0.5 | 14 | 4 |
| 1 μg IR61, 3 μg SG88, 3 μg SG132, 3 μg SG120 | 1350 | no | 0.5 | 14 | 3 |
| 1 μg IR61, 9 μg SG120 | 1350 | no | 0.5 | 14 | 3 |
| 1 μg IR62, 3 μg SG88, 3 μg SG132, 3 μg SG120 | 1100 | no | 0.5 | 14 | 7 |
| 1 μg IR62, 9 μg SG120 | 1100 | no | 0.5 | 14 | 10 |
| 1 μg IR63, 3 μg SG88, 3 μg SG132, 3 μg SG120 | 1100 | no | 0.5 | 14 | 11 |
| 1 μg IR63, 9 μg SG120 | 1100 | no | 0.5 | 14 | 6 |

| Bombardment Set 2 | | | | | | |
| --- | --- | --- | --- | --- | --- | --- |
| **DNA MIXTURE** | **GOLD BEADS** | **RUPTURE**  **DISKS (psi)** | **DNA**  **LINEARIZATION** | **V worms (ml)** | **N (selection**  **plates)** | **N (min. independent**  **transmitting strains)** |
| 10 μg IR89 | 0.6 μm | 1100 | PciI | 0.8 | 12 | 0 |
| 10 μg IR89 | 0.6 μm | 1350 | PciI | 0.8 | 12 | 0 |
| 10 μg IR89 | 1 μm | 1100 | PciI | 0.8 | 12 | 6 |
| 10 μg IR89 | 1 μm | 1350 | PciI | 0.8 | 12 | 4 |
| 10 μg IR89 | 0.3 μm - 3 μm | 900 | PciI | 0.8 | 12 | 8 |
| 10 μg IR89 | 0.3 μm - 3 μm | 1100 | PciI | 0.8 | 12 | 10 |

**Table S6.**

| **DNA MIXTURE** | **RUPTURE**  **DISKS (psi)** | **DNA**  **LINEARIZATION** | **V worms (ml)** | **N (selection**  **plates)** | **N (min. independent**  **transmitting strains)** |
| --- | --- | --- | --- | --- | --- |
| 1 μg SG238, 3 μg SG132, 6 μg SG120 | 1100 | no | 1.2 | 30 | 10 |
| 1 μg SG238, 3 μg SG132, 6 μg SG120 | 1100 | SfoI, NotI (DNA not purified afterwards, mixture directly coated onto the beads) | 1.2 | 30 | 23 |
| 1 μg SG238, 3 μg SG132, 6 μg SG120 | 1100 | SfoI, NotI (DNA purified afterwards, then coated onto the beads) | 1.2 | 30 | 28 |

**Supplementary Note**

When treating wild-type *C. elegans* at different larval stages, we have noticed a decreasing effect of the agent on later developmental stages. When treated as L1s, worms are incapable of development and arrest at L1 or L2 (L3 at lower hygromycin B concentrations) and eventually die. We observed this effect even for very high worm densities and hygromycin B concentrations lower than our standard 0.3 mg ml^-1^. We never found adult worms in samples treated at L1, even 10 days after addition of antibiotic. In contrast, when treated as L4s, worms showed slowed development and delayed egg-laying, but eventually reached adulthood and produced progeny, even at lower worm densities. However, their progeny arrested at L1 or L2 and never gave rise to new populations.
